# Author Correction: The oomycete *Lagenisma coscinodisci* hijacks host alkaloid synthesis during infection of a marine diatom

**DOI:** 10.1038/s41467-020-15527-y

**Published:** 2020-03-31

**Authors:** Marine Vallet, Tim U. H. Baumeister, Filip Kaftan, Veit Grabe, Anthony Buaya, Marco Thines, Aleš Svatoš, Georg Pohnert

**Affiliations:** 1 Research Group Plankton Community Interaction, Max Planck Institute for Chemical Ecology, Jena, Germany; 2 Research Group Mass Spectrometry/Proteomics, Max Planck Institute for Chemical Ecology, Jena, Germany; 3 Research Group Olfactory Coding, Department of Evolutionary Neuroethology, Max Planck Institute for Chemical Ecology, Jena, Germany; 4 Senckenberg Biodiversity and Climate Research Centre, Frankfurt am Main, Germany; 5 Department of Biological Sciences, Institute for Ecology, Evolution and Diversity, Goethe University, Frankfurt am Main, Germany; 6 Bioorganic Analytics, Institute for Inorganic and Analytical Chemistry, Friedrich Schiller University, Jena, Germany; 7 Microverse Cluster, Friedrich Schiller University Jena, Neugasse 23, 07743 Jena, Germany

**Keywords:** Chemical ecology, Cellular microbiology, Water microbiology, Pathogens

Correction to: *Nature Communications* 10.1038/s41467-019-12908-w, published online 30 October 2019.

The original version of this article contained an error in the chemical nomenclature of compound 4-CTC in the abstract and the results section, which incorrectly read ‘4-carboxy-2,3,4,9-tetrahydro-1H-β-carboline’. The correct name for compound 4-CTC according to IUPAC nomenclature is ‘2,3,4,9-tetrahydro-1H-β-carboline-3-carboxylic acid’. This has been corrected in the PDF and HTML versions of the article.

