# Supplementary material for: The oomycete Lagenisma coscinodisci hijacks host alkaloid synthesis during infection of a marine diatom
Source: Nat Commun. 2019 Oct 30;10:4938. doi: 10.1038/s41467-019-12908-w (PMC6821873; doi:10.1038/s41467-019-12908-w)
Supplement: Supplementary file 1 — Supplementary Information [file 41467_2019_12908_MOESM1_ESM.pdf]

Supplementary information

The oomycete *Lagenisma coscinodisci* hijacks host -alkaloids synthesis during infection of a marine diatom

Vallet et al.

Supplementary Table 1. Infection assessment of cells treated with filtrate from infected cultures (treatment) and untreated cells (control) over 4 days of incubation. The average infection rate (mean) and standard error (SE) were determined for biological triplicates (n = 3).

	Control		Treatment	
	Mean (%)	SE	Mean (%)	SE
Day 1	5.1	0.4	17.1	1.8
Day 2	12.9	0.6	22.4	2.1
Day 3	34.4	11.0	47.9	3.4
Day 4	92.8	2.4	92.1	2.5

Supplementary Table 2. Sample list with running order of the LC-MS sequence. Cell density was determined in a Sedgewick Rafter counting chamber. CG: *C. granii*. The average cell count was 316, 24 and 354 for exposed (n = 6), infected (n = 6) and healthy (n = 12) conditions, respectively. ANOVA on ranks found a significant difference between the cell count of the treatment (H=18,405 dF= 2, p-value < 0.001). The Dunn's procedure to compare multiple groups showed that infected vs healthy cell counts differed statistically (difference of ranks = 14, Q = 4, p-value < 0.05).

Sequence order	Sample name	Condition	Co-culture chamber Number	Cell density (cells.mL^{-1})	Normalization factor
1	blank_methanol				
2	20171130_ma_f2	Guillard's medium (f/2)			
3	blank_methanol_2				
4	blank_standard_mix				
5	20171204_ma_3-4b	Healthy CG	cc4	498	1.42
6	20171204_ma_3-1b	Healthy CG	cc1	529	1.34
7	blank_methanol_3				
8	20171204_ma_3-7b	Infected CG	cc7	38	18.61
9	20171204_ma_3-11a	Healthy (exposed) CG	cc11	331	2.14
10	blank_methanol_4				
11	qc_1				
12	20171204_ma_3-4a	Healthy CG	cc4	346	2.04
13	20171204_ma_3-10b	Infected CG	cc10	42	16.83
14	blank_methanol_5				
15	20171204_ma_3-10a	Healthy (exposed) CG	cc10	213	3.32
16	20171204_ma_3-11b	Infected CG	cc11	54	13.09
17	blank_methanol_6				
18	qc_2				
19	20171204_ma_3-12a	Healthy (exposed) CG	cc12	324	2.18
20	20171204_ma_3-9b	Infected CG	cc9	57	12.40
21	blank_methanol_7				
22	20171204_ma_3-8b	Infected CG	cc8	25	28.28

23	20171204_ma_3-9a	Healthy (exposed) CG	cc9	309	2.29
24	blank_methanol_8				
25	qc_3				
26	20171204_ma_3-5a	Healthy CG	cc5	363	1.95
27	20171204_ma_3-6a	Healthy CG	cc6	372	1.90
28	blank_methanol_9				
29	20171204_ma_3-5b	Healthy CG	cc5	511	1.38
30	20171204_ma_3-12b	Infected CG	cc12	24	29.46
31	blank_methanol_10				
32	qc_4				
33	20171204_ma_3-8a	Healthy (exposed) CG	cc8	235	3.01
34	20171204_ma_3-2a	Healthy CG	cc2	707	1.00
35	blank_methanol_11				
36	20171204_ma_3-7a	Healthy (exposed) CG	cc7	328	2.16
37	20171204_ma_3-3a	Healthy CG	cc3	607	1.16
38	blank_methanol_12				
39	qc_5				
40	20171204_ma_3-2b	Healthy CG	cc2	561	1.26
41	20171204_ma_3-1a	Healthy CG	cc1	320	2.21
42	blank_methanol_13				
43	20171204_ma_3-6b	Healthy CG	cc6	351	2.01
44	20171204_ma_3-3b	Healthy CG	cc3	641	1.10
45	blank_methanol_14				
46	blank_methanol_15				

Supplementary Table 3. Suggested structures of the up-regulated metabolites in infected cells, organized by their retention time (RT in minute). The metabolites are elucidated by HR-MS, MS^2 and library comparison. Metabolites with proven identity by comparison with analytical standards are indicated with (*). LysoPC: lysophosphatidylocholine.

RT	$[M+H]^+$ Observed mass	Deviation (ppm)	Compound chemical formula	Metabolite	Identifier CHEBI number	Diagnostic fragments (*m/z*)
1.1	274.1869	1.5	$C_{11}H_{23}N_5O_3$	Arginyl-valine	CHEBI:73823	215.0526, 70.0651
1.3	338.1819	0.9	$C_{15}H_{23}N_5O_4$	Tyrosyl-arginine	CHEBI:17537	175.1190, 58.1190, 112.0867
1.4	268.1036	1.5	$C_{10}H_{13}N_5O_4$	Adenosine*	CHEBI:16335	136.0616
2.4	322.1869	1.2	$C_{15}H_{23}N_5O_3$	Arginyl-phenylalanine	CHEBI:73818	215.0526, 70.0651
2.7	232.1401	1.3	$C_8H_{17}N_5O_3$	Glycyl-arginine	CHEBI:73860	158.0922, 16.0704, 112.0866
3.2	217.0969	1.2	$C_{12}H_{12}N_2O_2$	4-CTC*	CHEBI:91151	144.0804, 84. 9595
3.2	169.0758	1.2	$C_{11}H_8N_2$	β-carboline*	CHEBI:109895	142.0649, 115.0541
3.8	260.1853	1.2	$C_{13}H_{25}NO_4$	Hexanoyl-carnitine	CHEBI:70749	243.1701, 85.0283
4.3	286.2009	1.0	$C_{15}H_{27}NO_4$	Octenoyl-carnitine	CHEBI:71012	85.0283
6.4	542.3225	3.0	$C_{28}H_{48}NO_7P$	Lyso:PC (20:5)	CHEBI:88686	483.2480, 39.2813, 184.0733, 46.9817, 104.1069, 86.0963, 60.0809
6.5	494.3231	2.0	$C_{24}H_{48}NO_7P$	Lyso:PC (16:1)	CHEBI:64560	476.3134, 84.0731, 104.1068, 86.0963, 60.0809
6.6	280.263	1.4	$C_{18}H_{33}NO$	Linoleamide*	CHEBI:82984	263.2366, 245.2262
6.9	520.3392	1.0	$C_{26}H_{50}NO_7P$	Lyso:PC (18:2)*	CHEBI:64549	502.3394, 84.0731, 124.9996, 04.1068, 86.0963, 60.0809
6.9	568.3389	1.4	$C_{30}H_{50}NO_7P$	Lyso:PC (22:6)	CHEBI:64567	550.3278, 84.0732, 124.9997, 04.1068, 86.0963, 60.0809
6.9	544.3386	2.0	$C_{28}H_{50}NO_7P$	Lyso:PC (20:4)	CHEBI:64568	526.3297, 41.2847, 184.0733, 24.9998, 104.1069, 86.0964, 60.0810
7.2	233.1896	1.3	$C_{16}H_{24}O$	Ionone	CHEBI:49248	177.1274, 21.1011, 93.0698
7.3	570.3543	1.9	$C_{30}H_{52}NO_7P$	Lyso:PC (22:5)	CHEBI:74349	552.3448, 84.0731, 124.9997, 104.1068, 86.0963, 60.0809
7.4	468.3077	1.5	$C_{22}H_{46}NO_7P$	Lyso:PC (14:0)	CHEBI:64483	450.3088, 84.0731, 104.1068, 86.0962, 58.0653
9.9	581.3978	1.9	$C_{40}H_{52}O_3$	Phoenicoxanthin	CHEBI:80216	563.3884, 109.1011

Supplementary Table 4. Concentration of β-carboline and 4-CTC determined in the exudates of infected and healthy cultures of *C. granii* after solid phase extraction and quantification by UHPLC-HR-MS-MS.

Sample code	Intensity for β-carboline (a.u.)	Concentration of β-carboline (pM)	Intensity for 4-CTC (a.u.)	Concentration of 4-CTC (pM)
20190803_CG_exudates_healthy_MV13	5.13E+05	<18.4	2.38E+04	<21.5
20190803_CG_exudates_healthy_MV14	7.42E+05	<18.4	8.20E+04	<21.5
20190803_CG_exudates_healthy_MV15	5.88E+05	<18.4	5.22E+04	<21.5
20190803_CG_exudates_infected_MV10	2.75E+06	<18.4	1.49E+05	<21.5
20190803_CG_exudates_infected_MV11	5.28E+06	47.3	1.21E+05	<21.5
20190803_CG_exudates_infected_MV12	5.98E+06	55.9	8.02E+04	<21.5

Supplementary Figure 1. Growth of cells treated with filtrate of the infected cultures (treatment) and cells grown in control medium (control). Error bars indicate the standard error of mean.

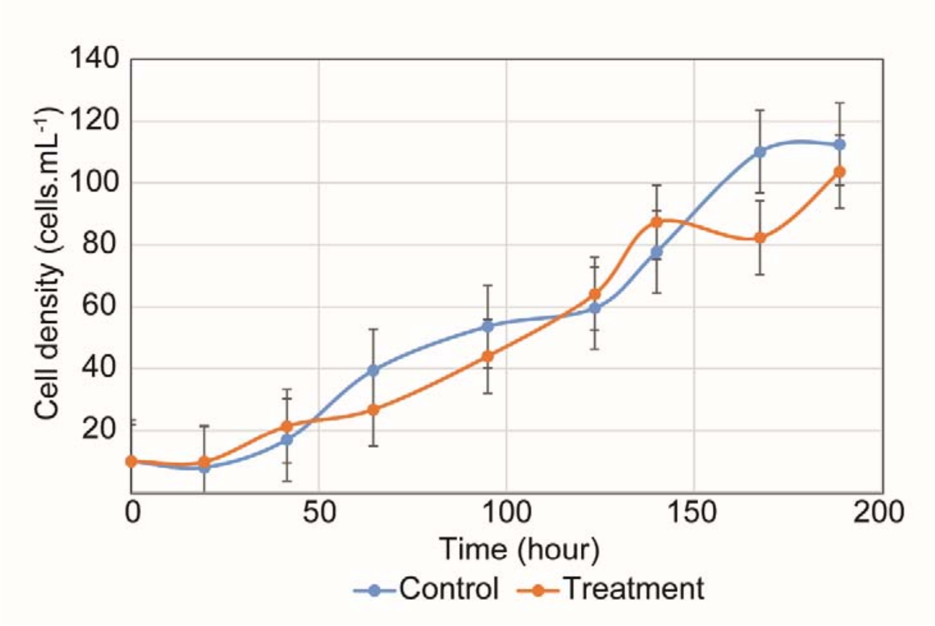

Supplementary Figure 2. a) Experimental setup depicting *C. granii* shells in methanol suspension which were deposited on HTC printed microscope glass slides, with highlights of surface area and shell area (left picture) and shells after laser abrasion (right picture). The intensity for β-carboline *m*/*z* 169.0760 detected at 1 fg deposited for 26 diatom shells was 27.69 a.u. SD±38.03 (referred in the Source Data file). The calculated limits of detection and quantification for β-carboline were 3.4 and 10.2 fg per shell, respectively. b) MALDI-HR-MS spectra of a single cell of *C. granii* infected with oomycete *L. coscinodisci* and zooming into the *m*/*z* range 100 to 170 allows assigning the M+H$^+$ and characteristic fragment ions of β-carboline in the infected cell.

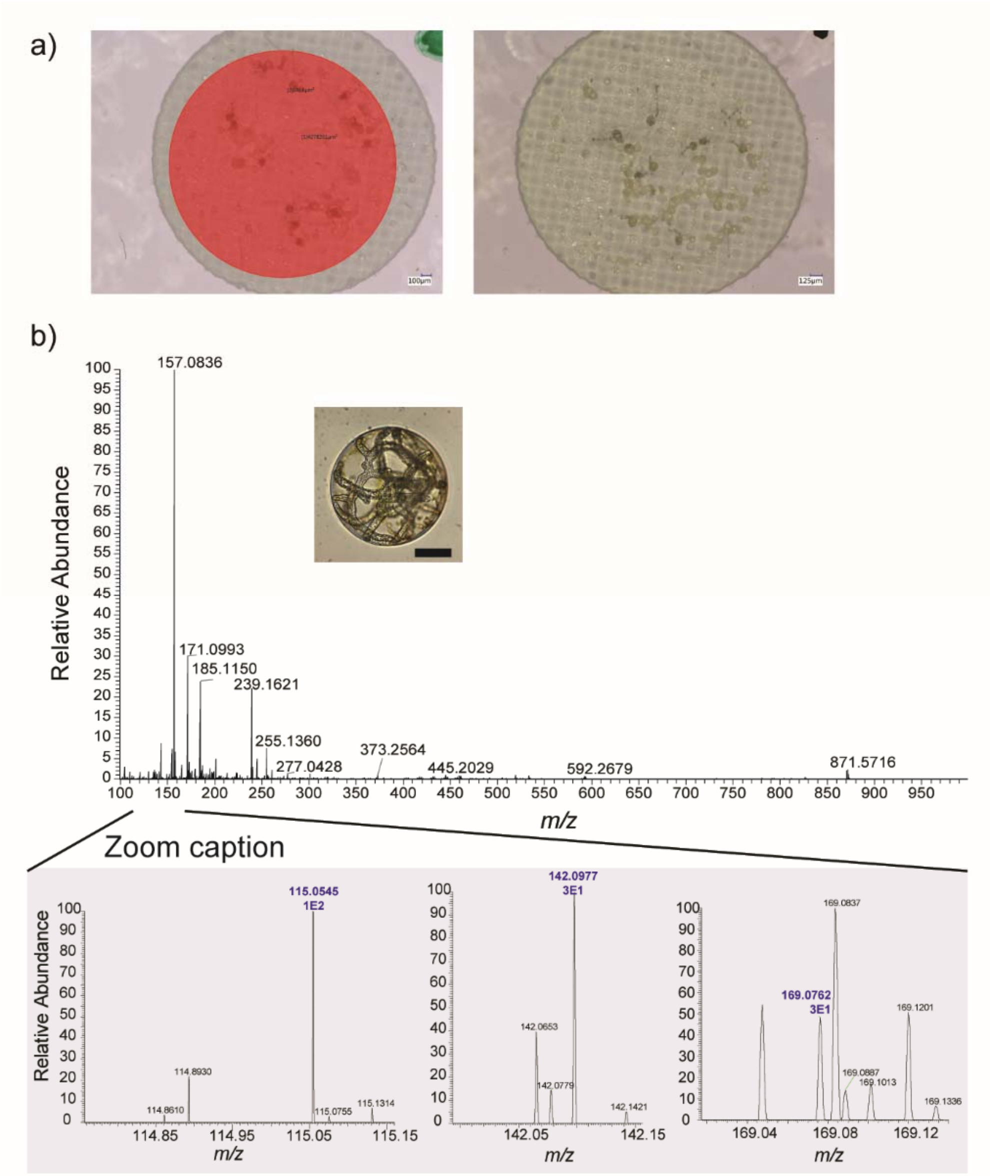

Supplementary Figure 3. Calibration curves for a) 4-CTC and b) β-carboline determined for absolute quantification with UHPLC-HR-MS/MS.

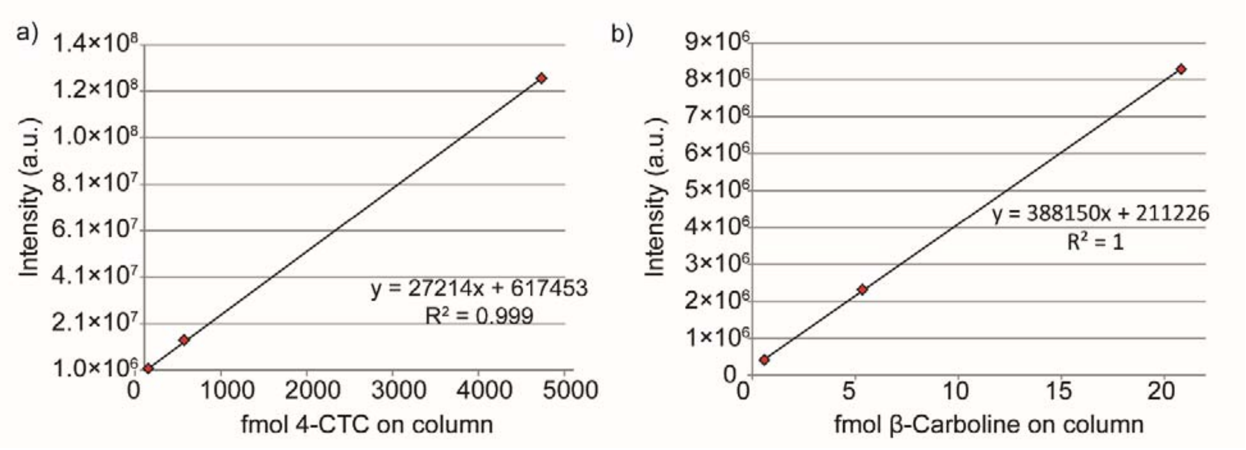

Supplementary Figure 4. Representative fluorescence spectra of the analytical standards chlorophyll *a*, β-carboline and 4-CTC recorded at an excitation of 405 nm.

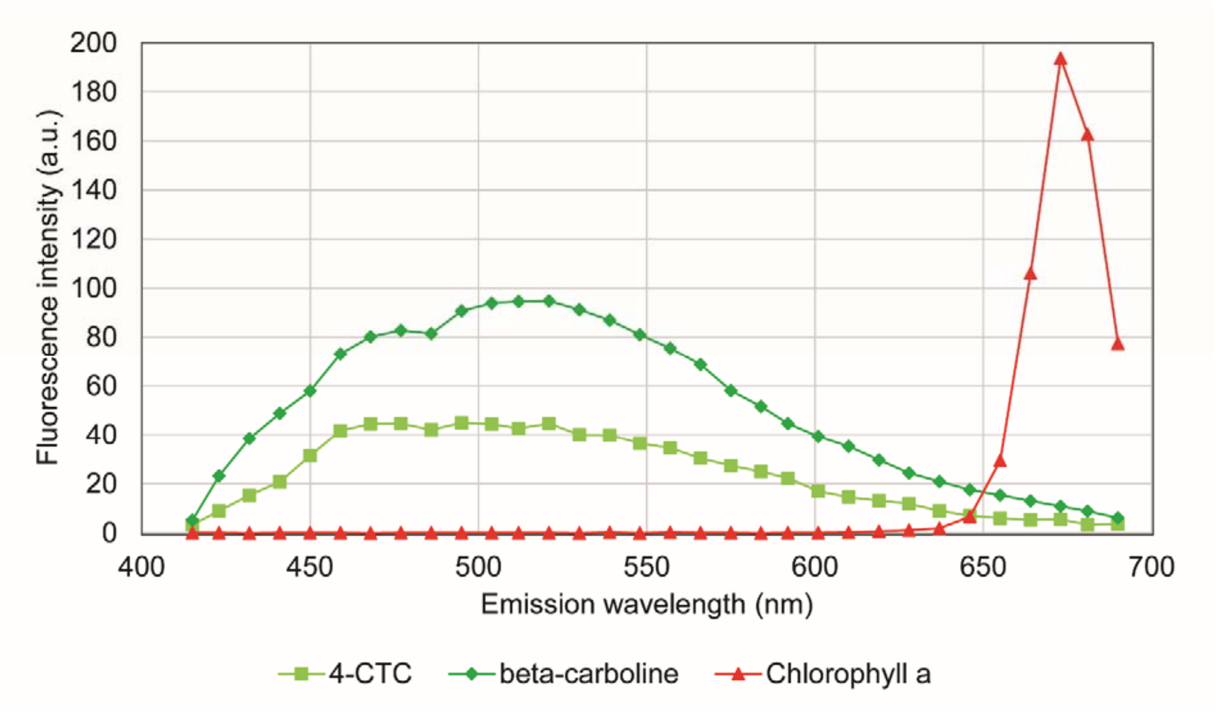

Supplementary Figure 5. Quantification by UHPLC-HR-MS/MS for a) 4-CTC and b) β-carboline determined after addition to axenic f/2 medium and extraction with solid phase extraction.

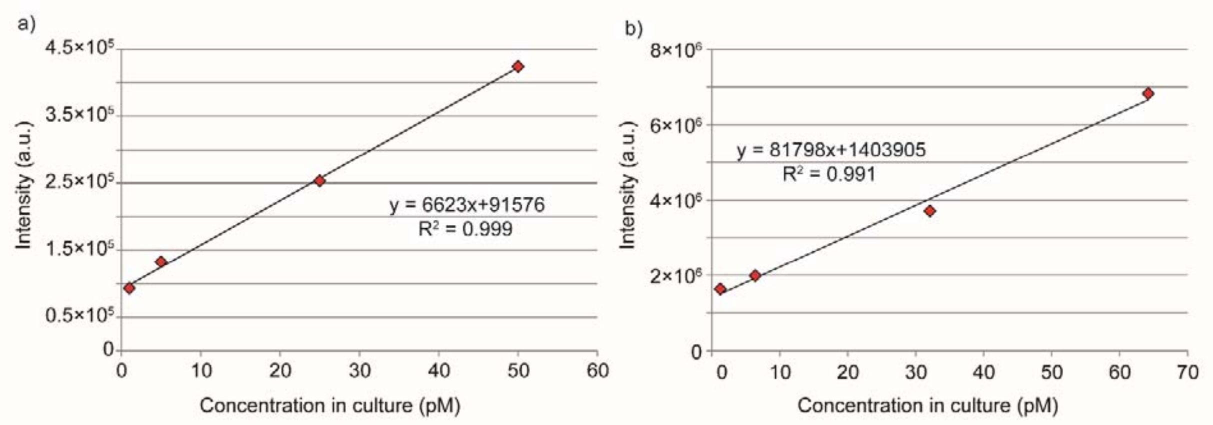

Supplementary Figure 6. MS/MS spectra, structure and ANOVA results for the identified metabolite 4-CTC a) in infected cell extract b) from analytical standard. The box-plots are displayed with the maximum, minimum, median lines and first/third quartiles and the samples as points.

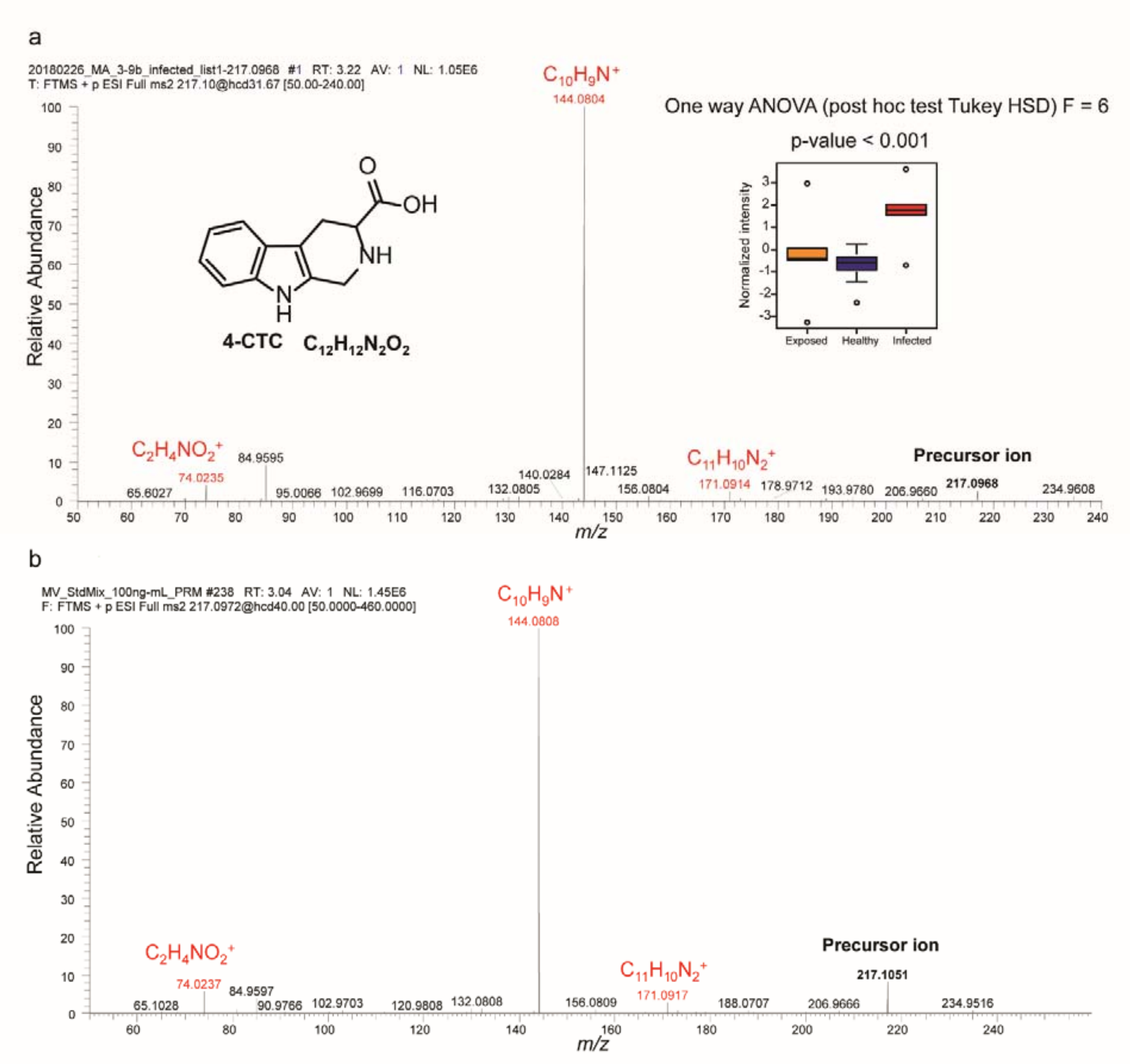

Supplementary Figure 7. MS/MS spectra, structure and ANOVA results for the identified metabolite β-carboline a) in infected cell extract b) from analytical standard. MS/MS characteristic fragments are supported by Crotti et al.[1] The box-plots are displayed with the maximum, minimum, median lines and first/third quartiles and the samples as points.

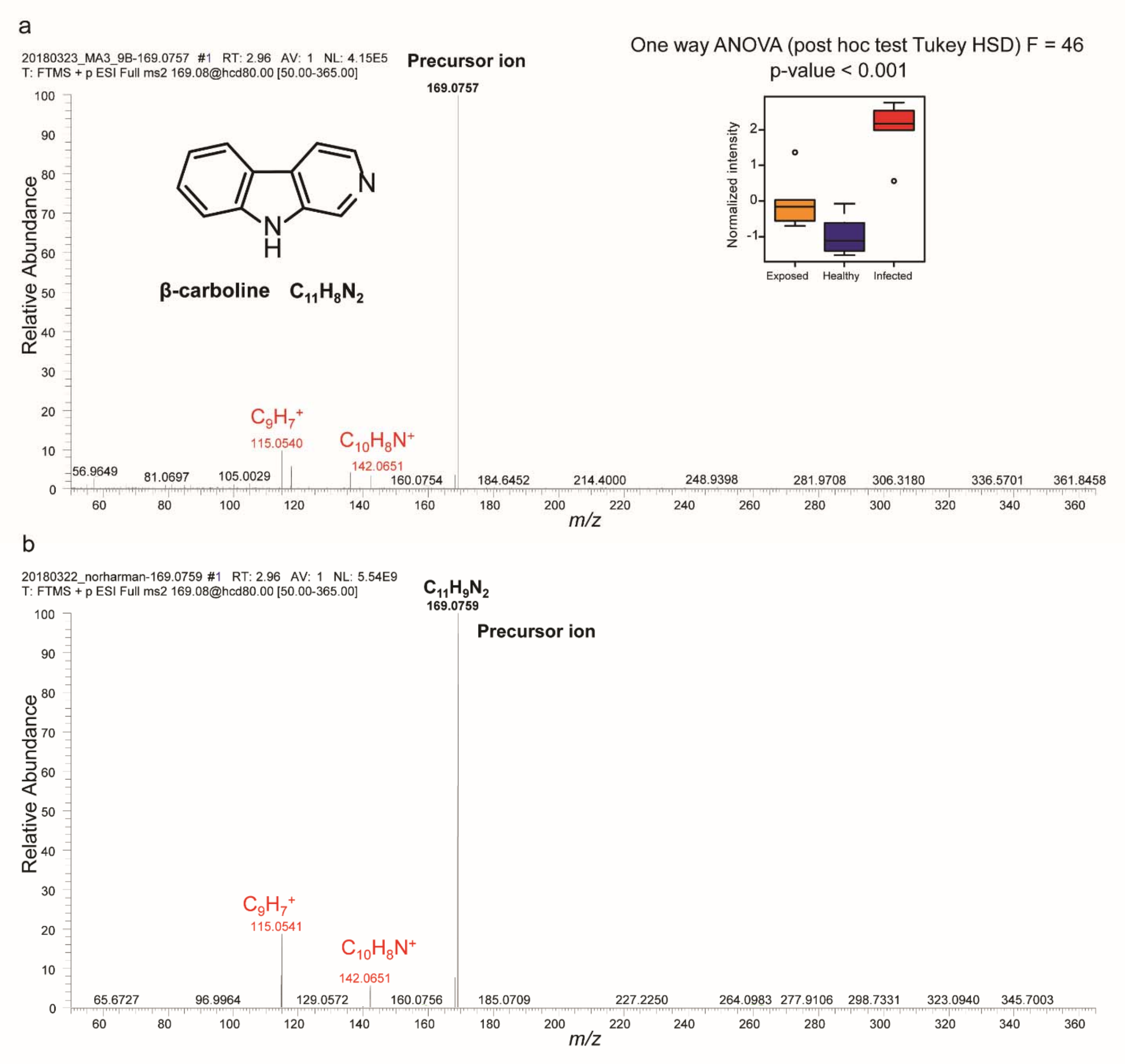

Supplementary Figure 8. MS/MS spectra, structure and ANOVA results for the identified metabolite linoleamide a) in infected cell extract b) from analytical standard. The box-plots are displayed with the maximum, minimum, median lines and first/third quartiles and the samples as points.

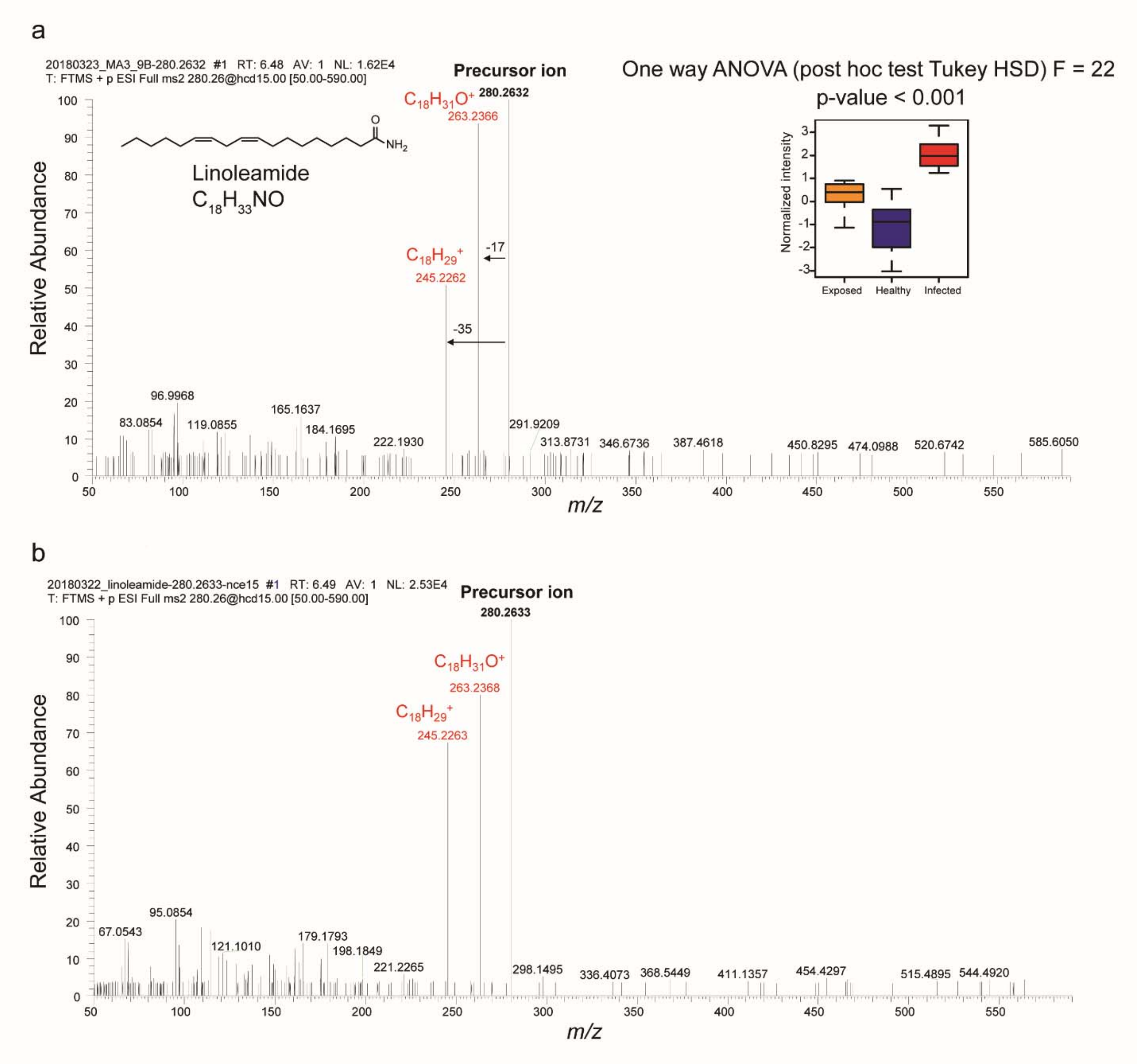

Supplementary Figure 9. MS/MS spectra, structure and ANOVA results for the identified dipeptides a) Arginyl-Phenylalanine b) Tyrosyl-arginine c) Arginyl-valine d) Glycyl-arginine. The box-plots are displayed with the maximum, minimum, median lines and first/third quartiles and the samples as points.

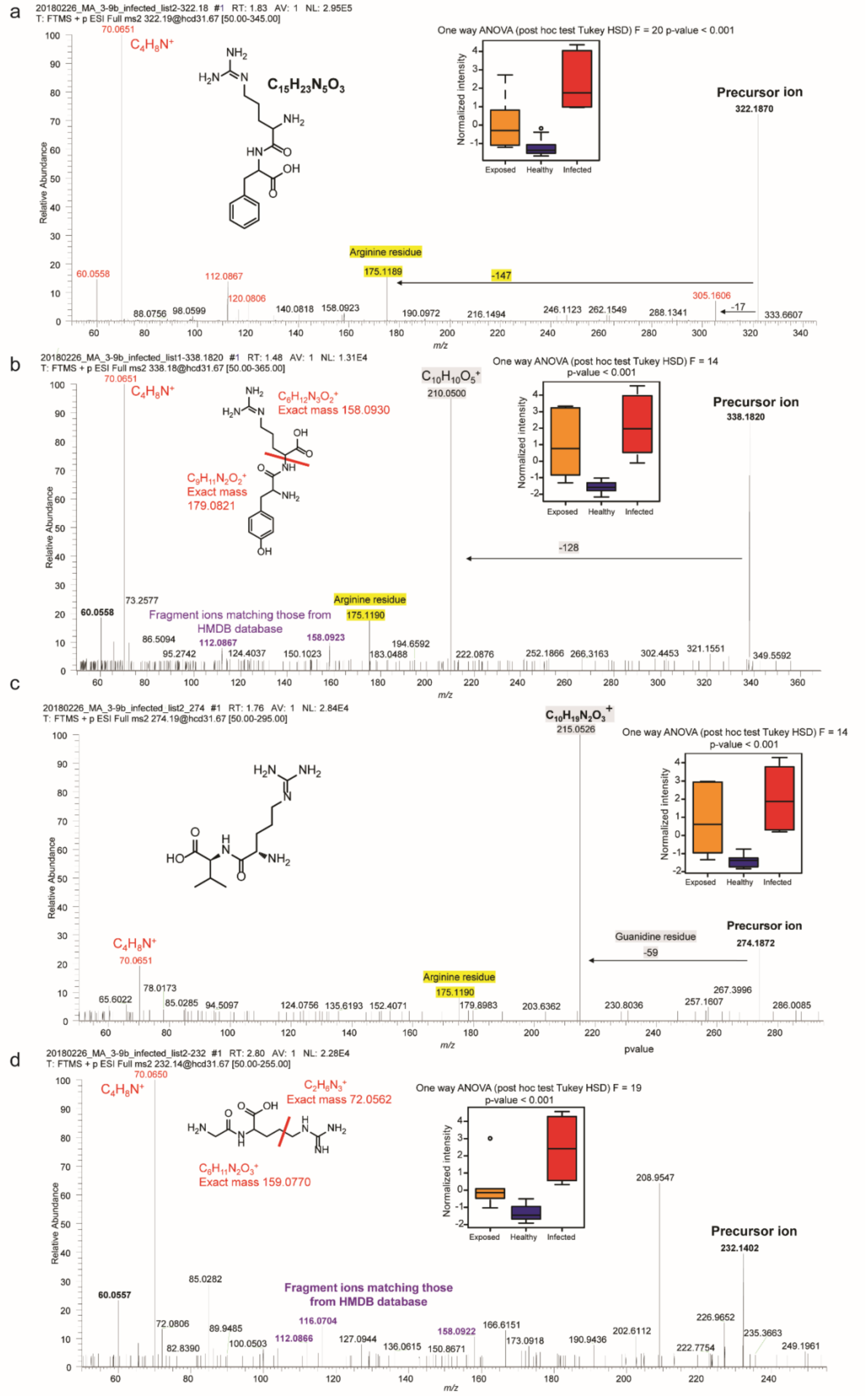

Supplementary Figure 10. MS/MS spectra and ANOVA result for the identified lysophosphatidylcholine a) in infected extract b) from analytical standard. MS/MS characteristic fragments are supported by Suarez-Garcia et al..[2] The box-plots are displayed with the maximum, minimum, median lines and first/third quartiles and the samples as points.

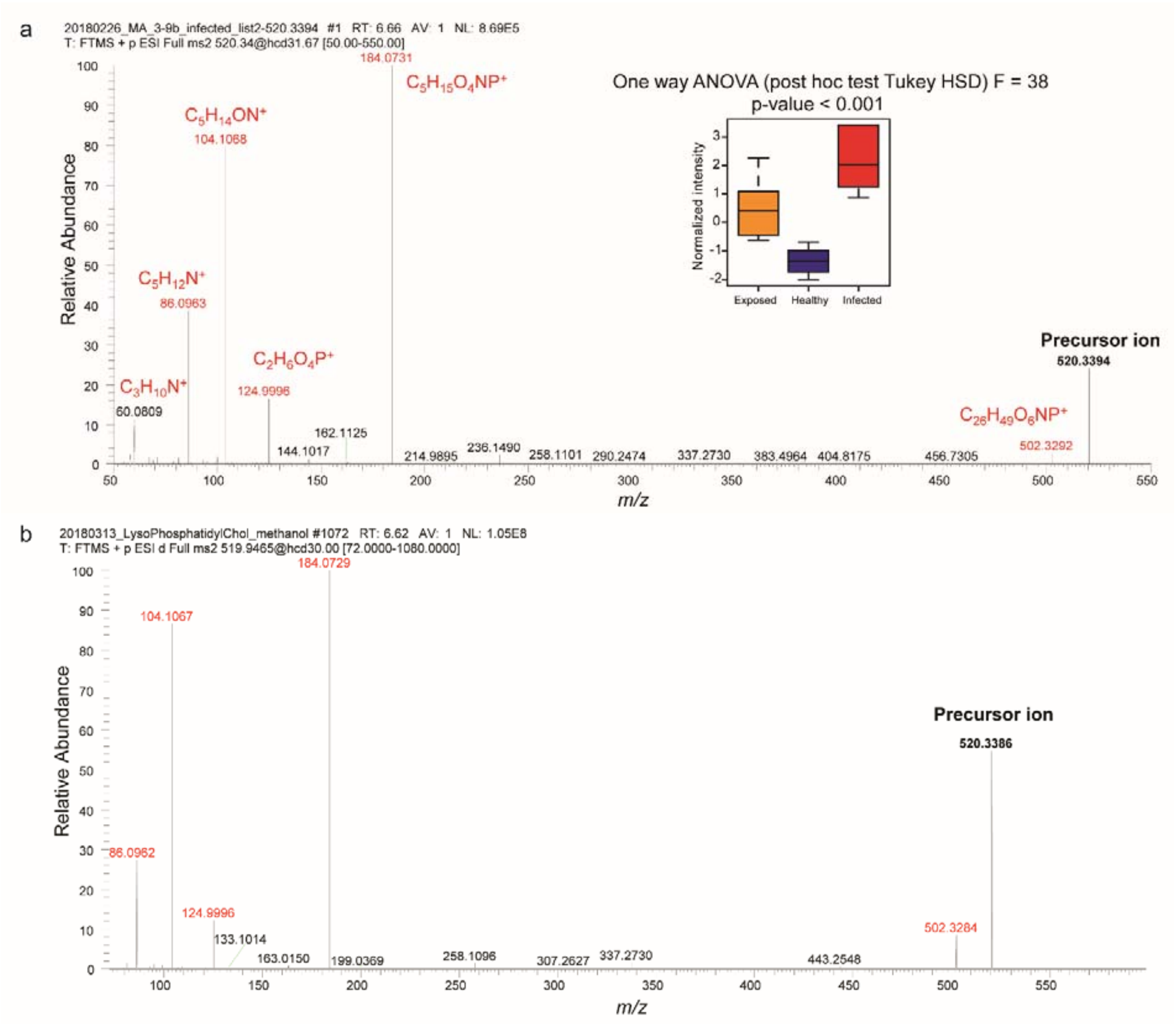

Supplementary Figure 11. MS/MS spectra from infected extract and ANOVA results for a) hexanoylcarnitine b) octanoylcarnitine. MS/MS characteristic fragments are supported by Cho et al..[3] The box-plots are displayed with the maximum, minimum, median lines and first/third quartiles and the samples as points.

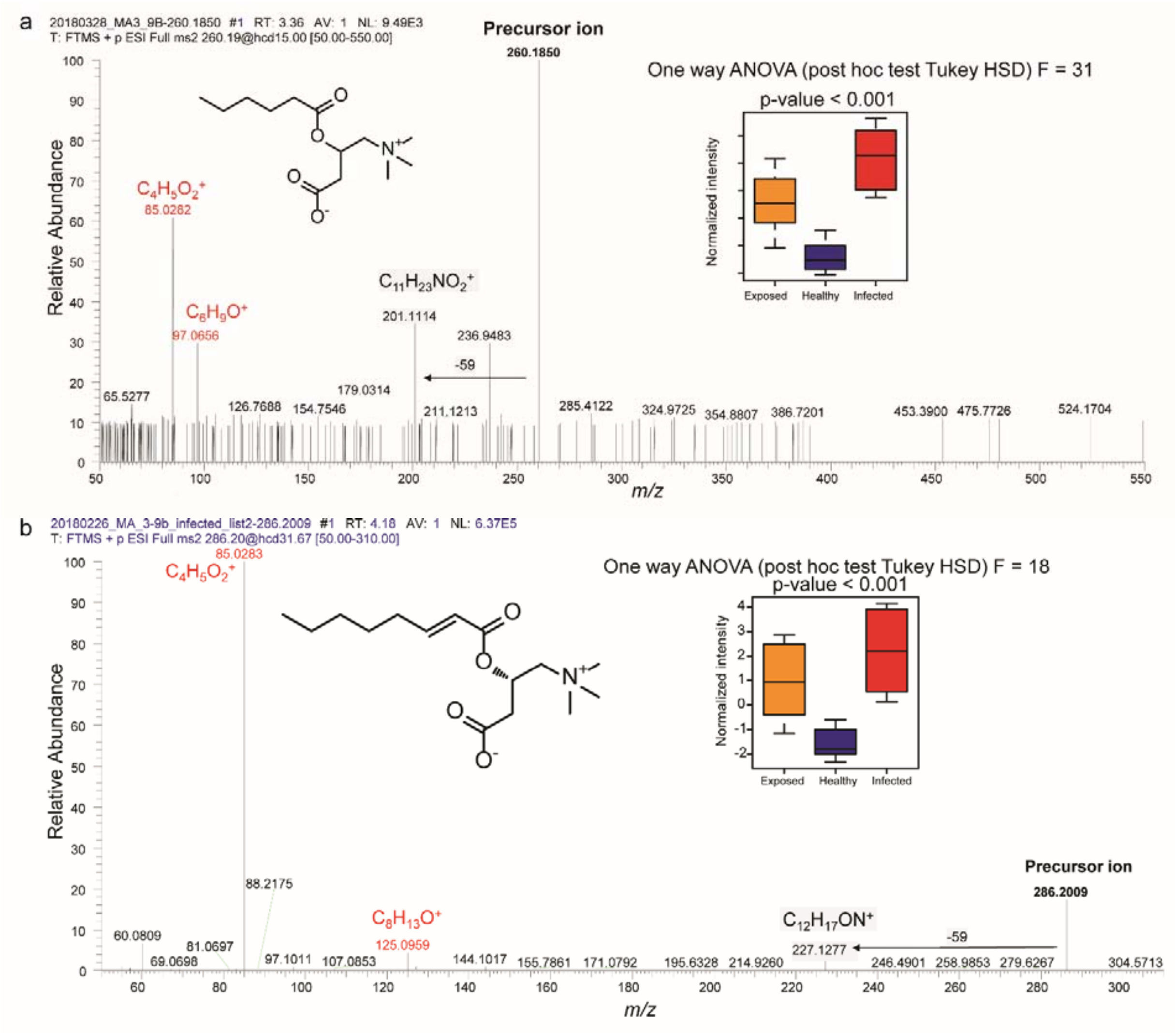

Supplementary Figure 12. MS/MS spectra from infected extract and ANOVA results for Lysophosphatidylcholines a) 18:2 b) 14:0 c) 16:1 d) 20:5. MS/MS characteristic fragments are

supported by Suárez-García et al., Development and validation of a UHPLC-ESI-MS/MS method for the simultaneous quantification of mammal lysophosphatidylcholines and lysophosphatidylethanolamines in serum.[4] The box-plots are displayed with the maximum, minimum, median lines and first/third quartiles and the samples as points.

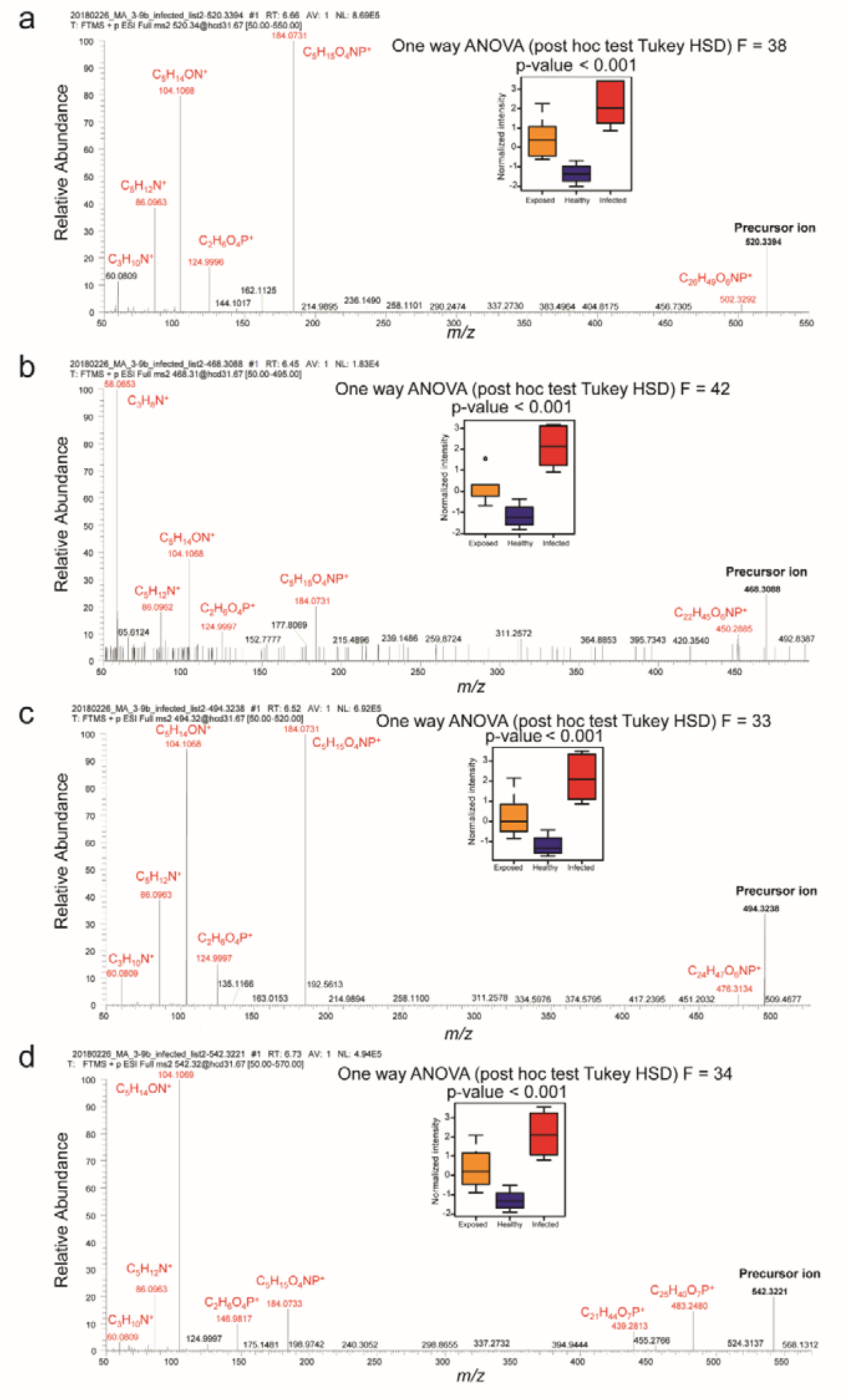

Supplementary Figure 13. MS/MS spectra from infected extract and ANOVA results for Lysophosphatidylcholines a) 20:4 b) 22:6 c) 22:5. The box-plots are displayed with the maximum, minimum, median lines and first/third quartiles and the samples as points.

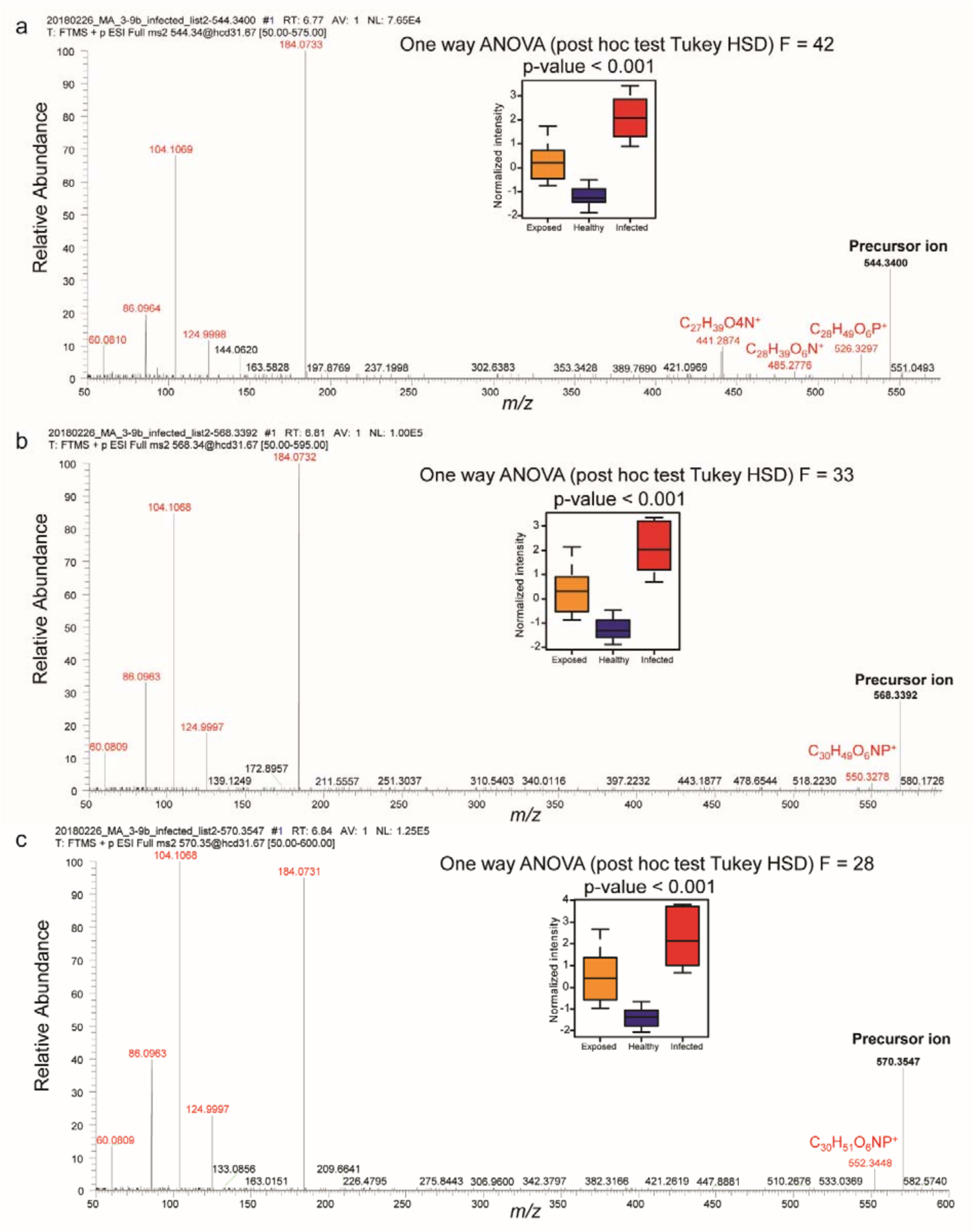

Supplementary Figure 14. a) MS and b) MS/MS spectra from infected extract with ANOVA result for the metabolite $C_{10}H_7Cl_2N$. The box-plots are displayed with the maximum, minimum, median lines and first/third quartiles and the samples as points.

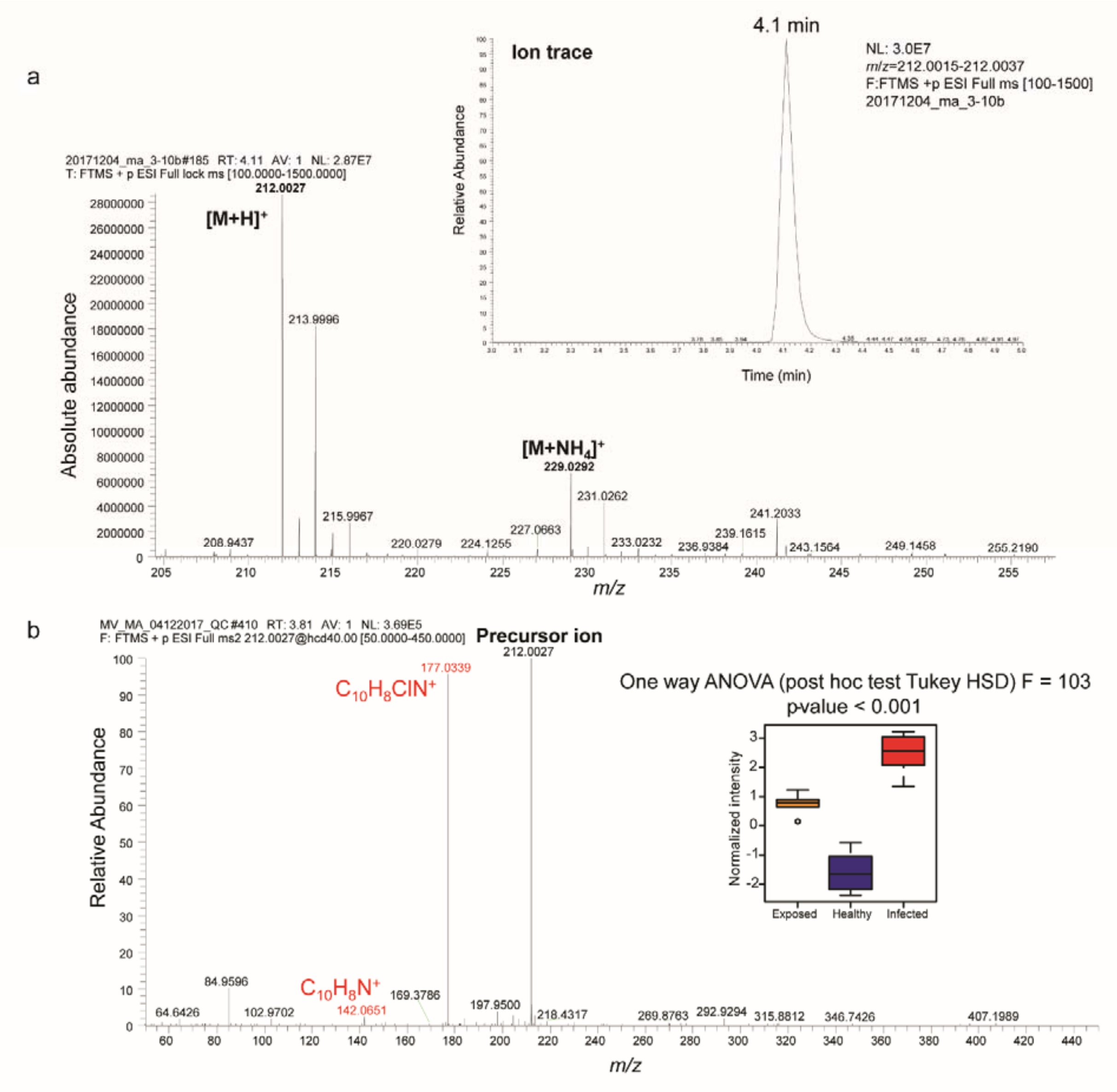

Supplementary References

1 Crotti, A. E., Gates, P. J., Lopes, J. L. & Lopes, N. P. Electrospray MS-based characterization of β-carbolines – mutagenic constituents of thermally processed meat. *Molecular Nutrition & Food Research* 54: 433-439 (2010)

2 Suárez-García, S., Arola, L., Pascual-Serrano, A., Arola-Arnal, A., Aragones, Bladé, C., Suárez, M. Development and validation of a UHPLC-ESI-MS/MS method for the simultaneous quantification of mammal lysophosphatidylcholines and lysophosphatidylethanolamines in serum *J. Chrom. B* 1055-1056: 86-97 (2017).

3 Cho, S.-H., Lee J., Lee W. Y., Chung B. C. Direct determination of acylcarnitines in amniotic fluid by column - switching liquid chromatography with electrospray tandem mass spectrometry *Rapid Commun. Mass Spectrom.* **20**, 1741-1746 (2006).

4 Suárez-García, S. Arola, L., Pascual-Serrano, A., Arola-Arnal, A., Aragones, G., Bladé, C., Suárez, M. Development and validation of a UHPLC-ESI-MS/MS method for the simultaneous quantification of mammal lysophosphatidylcholines and lysophosphatidylethanolamines in serum. *J. Chrom. B* 1055–1056: 86-97 (2017).
