# Supplementary material for: The oomycete Lagenisma coscinodisci hijacks host alkaloid synthesis during infection of a marine diatom
Source: Nat Commun. 2019 Oct 30;10:4938. doi: 10.1038/s41467-019-12908-w (PMC6821873; doi:10.1038/s41467-019-12908-w)
Supplement: Supplementary file 3 — Description of Additional Supplementary Files [file 41467_2019_12908_MOESM3_ESM.pdf]

Description of Additional Supplementary Files

File Name: Supplementary Movie 1

Description: Release of Lagenisma coscinodisci’s zoospores through an exit tube. Video speed was increased by 8 times.
